# An Inertial Measurement Unit-Based Wireless System for Shoulder Motion Assessment in Patients with Cervical Spinal Cord Injury: A Validation Pilot Study in a Clinical Setting

**DOI:** 10.3390/s21041057

**Published:** 2021-02-04

**Authors:** Riccardo Bravi, Stefano Caputo, Sara Jayousi, Alessio Martinelli, Lorenzo Biotti, Ilaria Nannini, Erez James Cohen, Eros Quarta, Stefano Grasso, Giacomo Lucchesi, Gabriele Righi, Giulio Del Popolo, Lorenzo Mucchi, Diego Minciacchi

**Affiliations:** 1 Department of Experimental and Clinical Medicine, University of Florence, Largo Brambilla 3, 50134 Florence, Italy; erezjames.cohen@unifi.it (E.J.C.); equarta@unifi.it (E.Q.); stefano.grasso@stud.unifi.it (S.G.); diego.minciacchi@unifi.it (D.M.); 2 Department of Information Engineering, University of Florence, via di S. Marta 3, 50139 Florence, Italy; stefano.caputo@unifi.it (S.C.); sara.jayousi@pin.unifi.it (S.J.); alessio.martinelli@unifi.it (A.M.); lorenzo.biotti@unifi.it (L.B.); 3 Spinal Unit, Azienda Ospedaliero-Universitaria Careggi, Largo Piero Palagi 1, 50139 Florence, Italy; nanninii@aou-careggi.toscana.it (I.N.); lucchesig@aou-careggi.toscana.it (G.L.); righiga@aou-careggi.toscana.it (G.R.); delpopolog@aou-careggi.toscana.it (G.D.P.)

**Keywords:** inertial measurement unit, wireless sensors network, motion tracking, kinematics, range of motion, shoulder, goniometer, spinal cord injury, tetraplegia, clinical setting

## Abstract

Residual motion of upper limbs in individuals who experienced cervical spinal cord injury (CSCI) is vital to achieve functional independence. Several interventions were developed to restore shoulder range of motion (ROM) in CSCI patients. However, shoulder ROM assessment in clinical practice is commonly limited to use of a simple goniometer. Conventional goniometric measurements are operator-dependent and require significant time and effort. Therefore, innovative technology for supporting medical personnel in objectively and reliably measuring the efficacy of treatments for shoulder ROM in CSCI patients would be extremely desirable. This study evaluated the validity of a customized wireless wearable sensors (Inertial Measurement Units—IMUs) system for shoulder ROM assessment in CSCI patients in clinical setting. Eight CSCI patients and eight healthy controls performed four shoulder movements (forward flexion, abduction, and internal and external rotation) with dominant arm. Every movement was evaluated with a goniometer by different testers and with the IMU system at the same time. Validity was evaluated by comparing IMUs and goniometer measurements using Intraclass Correlation Coefficient (ICC) and Limits of Agreement (LOA). inter-tester reliability of IMUs and goniometer measurements was also investigated. Preliminary results provide essential information on the accuracy of the proposed wireless wearable sensors system in acquiring objective measurements of the shoulder movements in CSCI patients.

## 1. Introduction

Spinal cord injury (SCI) is a debilitating neurological condition which can result in a total or partial motor and sensory function impairment below the site of the injury, associated with a various degree of bladder, bowel and sexual dysfunctions [1]. The sensory and/or motor impairment is caused by the loss of communication between the brain areas devoted to motor/sensory processing and the axons of neural cells controlled by spinal cord levels below the injury site and innervating the body surface. The site and completeness of the injury largely determine the clinical outcomes of SCI [2]. Lesions at lower spinal segments (i.e., sacral, lumbar, or thoracic) cause the loss of motor and/or sensory function in lower limbs and trunk (paraplegia) while more rostral lesions (i.e., cervical) involve lower limbs, trunk, and upper limbs (tetraplegia).

Regarding severity, spinal cord injury can be graded on the basis of motor and sensory dysfunction as follows: no motor or sensory function is preserved; sensory function preserved but not motor function; both motor and sensory functions are—partially—preserved below the neurological level [3].

For CSCI individuals the residual motion of upper limbs is a key-element to perform activities of daily living and participate in community [4,5,6].

Shoulder range of motion (ROM) is involved in basic self-management skills (e.g., bathing, feeding or dressing), independent transferring skills (e.g., getting in/out of bed or moving to/from wheelchair) as well as sport and leisure activities participation [7].

In an attempt to increase functional independence of CSCI individuals, different surgical interventions and rehabilitation programs have been developed to restore deficits in motor control provoked by spinal cord injuries [8]. Therefore, tools to objectively and reliably measure the efficacy of a treatment process would be extremely desirable.

At the present moment, in clinical settings the assessment of the shoulder function in patients with cervical spinal cord injury (CSCI) is commonly limited to simple clinical observations by the examiner or based on rating scales, which are not only inefficient for detecting small differences but also inclined to produce subjective errors [9,10].

Alongside the observational examination, goniometer is a portable, inexpensive and handy tool traditionally used in clinical settings to assist doctors for the evaluation of shoulder movements [11]. However, conventional goniometric measurements can vary among testers [12,13] and require substantial time and effort in clinics.

In addition, clinical assessments using simple tools such as goniometer allow us to measure only the ROM and are not sufficient when attempting to assess complex patterns of upper limb movement in persons with CSCI. Thus, dynamic kinematic tests have been developed to integrate ROM evaluation while providing a more complete monitoring of upper limb functional movements [14,15].

Several movement analysis devices are available to assist objective and accurate kinematic measurements varying from simple video cameras to complex optical motion capture systems (e.g., [16,17,18,19]). Such evaluation systems generally demand highly expensive equipment, well-skilled personnel for operational procedure, as well as dedicated spaces, which is not realistically suitable for clinical settings in hospital or space-limited environments. This paved the way for the introduction of wearable, low-cost and user-friendly devices that can provide objective information of movement characteristics, with the possibility of monitoring these parameters in daily life, in accordance to future healthcare system [20,21].

In the last few years, a new generation of inertial measurement units (IMUs) has emerged, significantly impacting on motion tracking research due to their ease of use, relative low cost, and portability [22,23,24]. This technology, taking advantage of recent progress in miniature inertial sensors, consists of lightweight, non-invasive, wearable, wireless sensing units, which open a new opportunity to capture human motion in different settings [13,25].

An IMU device estimates orientation of human body segments based on data combined from multiple electromechanical sensors as accelerometers, tri-axial gyroscopes, and magnetometers through the use of sensor fusion algorithms such as a Kalman filter or complementary weighting algorithm [26,27,28].

The combination of information from different inertial sensor units is considered an advance in minimizing measurement errors of one component-based orientation (e.g., linear acceleration interference and inertial sensor drift), thus guaranteeing a more accurate and reliable estimation of motion [26,29,30,31]. Therefore, IMU systems have been increasingly adopted to efficiently measure joint ROM of several body parts such as lower limbs [32], trunk [33], as well as arm/shoulder [34,35,36].

In spite of the increasing application of IMU systems in shoulder joint measurements, only a few studies tested the validity of IMU in comparison with goniometer [13,37]. In [37] IMU and goniometer were compared by passively positioning the shoulder at specific angles and good concurrent validity of IMU was found, though they showed relatively low agreement at elevated shoulder positions. Also, in [13] an excellent agreement was exhibited between goniometer- and IMU-based measurements and the two methods were shown to be interchangeable for measuring active shoulder ROM. Overall, these investigations provided preliminary evidence of the reliability of IMUs in accurately measuring shoulder ROM by comparing it with a goniometer tool. Nonetheless, the tested populations in these studies consisted of healthy participants as the most of studies evaluating IMUs systems [38] and, as suggested by Rigoni et al. [13], their results might not be replicable in pathological individuals. It should be considered that the monitoring of active shoulder ROM in patients with CSCI can occur in a very different context if compared to healthy subjects. The introduction of a wheelchair in the setup is necessary when testing CSCI patients. However, this assistive device can interfere with the arm motion to be performed. Deviations from single plane of unrestricted movements were shown in a previous study to potentially influence the accuracy of technology used to measure shoulder ROM [39]. As such the wheelchair, along with deficits in motor control of upper limbs in this special population [40], could be a source of measurement error for IMU sensors when testing active shoulder movements. Moreover, electronic controls of motorized wheelchairs can reduce the accuracy of some components of IMU sensors (e.g., magnetometer). All these context-related factors could limit the generalization of the results reported by Rigoni to a specific population as the patients with CSCI while active shoulder movements are performed. Since IMU devices should ultimately support clinicians and patients suffering from pathological motor diseases, additional research is needed to understand the reliability of IMU systems on these special populations.

The objective of this pilot study was to investigate the validity of a customized, wireless wearable IMU-based sensors system in evaluating active shoulder movements in CSCI patients, while seated in a wheelchair, in a clinical setting. To achieve this, we compared the accuracy of the IMU system to goniometer method in measuring shoulder ROM during the performance of flexion, abduction, and extra- and intra-rotation movements of the upper limb. The IMU sensors used for this study were composed of an accelerometer and a tri-axial gyroscope without the implementation of the magnetometer, so as to reduce possible interference due to electronic controls in motorized wheelchairs. Also, according to previous studies investigating concurrent validity of IMU devices in monitoring ROM on pathological populations [39,41], our experimental design included an additional group of healthy subjects. In order to obtain uniform measurements between the different populations, even the healthy subjects performed the active shoulder movements while seated in a wheelchair. Finally, as it is important to know to what extent the measurement made by an instrument is dependent on the person (i.e., operator) who carries it out, in this study it was also investigated the levels of consistency between repeated goniometer measurements and repeated IMU measurements taken by different operators during the same movement.

## 2. Materials and Method

### 2.1. Shoulder Movements to Evaluate Range of Motion

**Figure 1:**
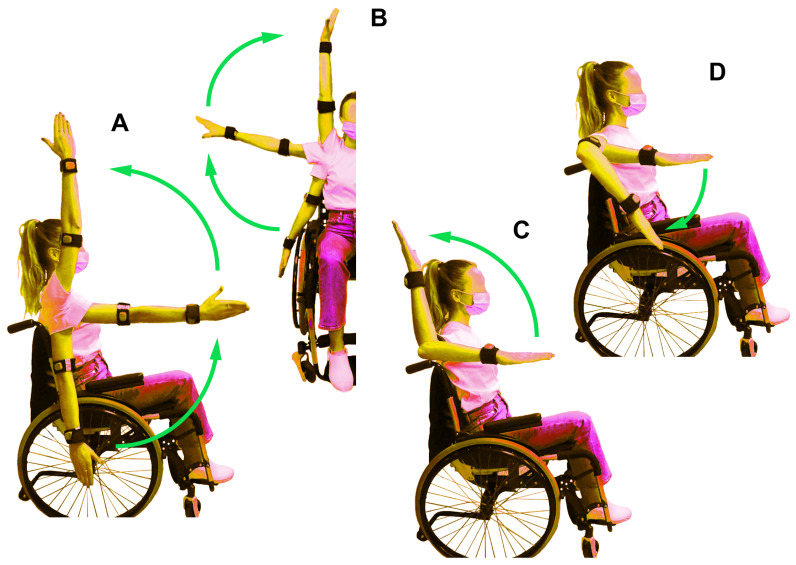
The four recorded active shoulder maneuvers: forward flexion (**A**), abduction (**B**), external rotation (**C**) and internal rotation (**D**).

This section describes the movements to evaluate shoulder ROM in individuals with spinal cord injury and healthy subjects. The tested movements and measurement procedures to evaluate shoulder ROM have been identified in forward flexion, abduction, external and internal rotation at 90° abduction (See Figure 1), following the study of Frye et al. [42].

For shoulder forward flexion, subject was asked to raise the arm straight up in front of him/her with the forearm in neutral position, i.e., the palm of the hand toward the mid-line (See Figure 1A). For shoulder abduction, the starting hand position was the same of flexion maneuver and the subject was required to raise the arm at the side (See Figure 1B).

For external and internal rotation at the 90° shoulder abduction, the shoulder and elbow were positioned in approximately 90° of abduction and flexion, respectively, with the forearm parallel to the floor.

For external rotation, subject was asked to rotate the forearm upwards, whereas in the internal rotation subject was required to rotate the forearm downwards (See Figure 1C,D). Scapular rotation was allowed during shoulder forward flexion and abduction. In addition, subjects were assisted by a clinician to keep the shoulder and the elbow at the initial position during external and internal rotation at the 90° shoulder abduction.

### 2.2. Goniometer Measurement Method

To measure the ROM for forward flexion, abduction, external and internal rotation at 90° of shoulder abduction (see Figure 1), a standard plastic goniometer (Gima Co., Gessate, Italy), characterized by two arms that align with the angle of the joint, was used to provide the degree of movement in that joint. The active movements of shoulder were calculated by identified landmarks (see Table 1). The methods used to measure ROM for each tested shoulder maneuver [39,43,44,45,46] are described below.

The flexion angle was calculated by lateral aspect of the glenohumeral joint and aligning its stationary arm parallel to the midline of the trunk and its moving arm with the lateral epicondyle of the humerus.

The abduction angle was calculated by placing the center fulcrum of goniometer on posterior aspect of the glenohumeral joint and aligning its stationary arm along the trunk (parallel to the spine) and its moving arm with the lateral epicondyle of the humerus.

The external and internal rotation angles at 90° of shoulder abduction were calculated by placing the center fulcrum of goniometer on olecranon process ulna, and aligning stationary arm parallel to the floor and its moving arm with the ulna styloid process.

### 2.3. Wearable Sensors System for Motion Assessment

**Figure 2:**
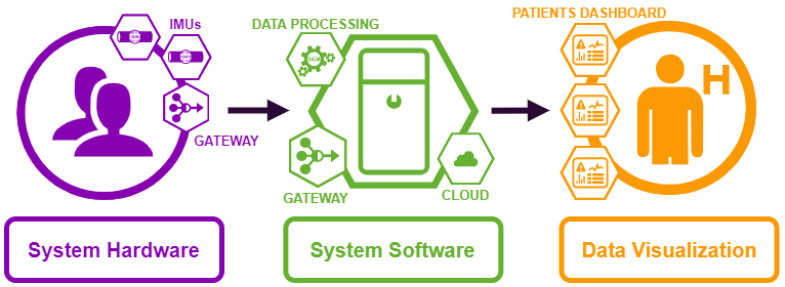
General scheme of the system consisting of 3 main modules (represented with different colors). The round element corresponds to an end user (human), while hexagonal elements (both hardware and software) corresponds to the system. In purple, the components of system hardware module; in green, the components of system software module and in orange, the components of data aggregation/visualization module.

A wearable sensors system was developed for motion assessment during the experimental tests. The system aims to create an innovative protocol for patient monitoring to be used both in hospital ward and/or in patient’s house. The system is composed of 3 main modules, as shown in Figure 2:

- **Hardware** module is the part of the system (for complete details see Appendix A.1) composed of IMU sensors and a gateway (Raspberry Pi);
- **Software** module is the part of the system (for complete details see Appendix A.3) composed of software components, which run on the gateway and provide the following functionalities: IMU sensors synchronization, data collection, and data processing to obtain the kinematics parameters used for medical evaluation of the movement (for complete details see Appendix A.2);
- **Data Visualization** module is the display part, showing data in real time to clinicians. Since this part is not necessary for the experimental campaign, it will be deployed as future development.

In Figure 2, the gateway symbol appears twice, in the hardware part and in the software part. In the hardware part it includes a communication protocol to synchronize the connection with the IMU sensors and collect the data. In the software part it includes a software program to process the data and store them into a cloud database for offline analysis.

Two sensors were used to measure shoulder ROM. The sensor on the arm was used to measure the angle during the flexion and abduction movements (Figure 1A,B), while the sensor on the wrist was used during the rotation movement at the 90° shoulder abduction (Figure 1C,D).

### 2.4. Experimental Campaign: Setup and Protocols

Experimental data were gathered in a room of the Careggi Hospital Spinal Unit, Florence. The four recorded active shoulder maneuvers (Figure 1) during the session consisted of: forward flexion, abduction, external and internal rotation at 90° of shoulder abduction [42]. The shoulder maneuvers were assessed on each participant’s dominant shoulder [47].

Each movement was performed by CSCI subjects sitting in their own wheelchair, as well as healthy controls who were provided with a wheelchair by the hospital [48]. This avoided possible differences in range of motion between the two groups due to potential interference with the wheelchair [48,49]. The wheelchair wheels were blocked during the tests execution [50].

At the beginning of the session, all participants were informed about the aim of the study and that they would perform 4 different active shoulder movements. After having signed the informed consent to participate in the experiment (See Section 2.4.2), they were fitted with the IMU-based system on the dominant arm.

**Figure 3:**
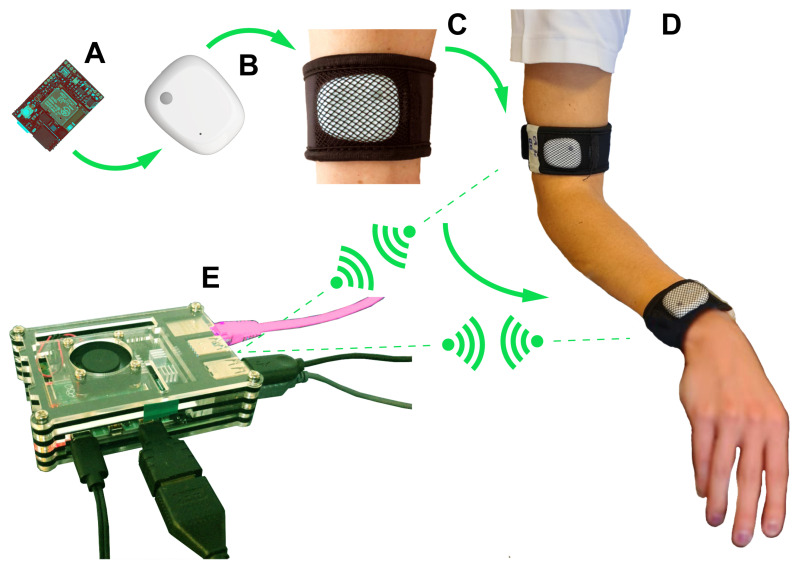
Wearable sensors system for motion assessment installation procedure: MetaMotionR (MMR) sensors boards (**A**) were put inside the cases (**A**→**B**); the cases were put in the velcro bands (**B**→**C**); the bands were placed as shown in (**D**); than the software on the Raspberry Gateway was run to collect data from sensors (**E**).

Two IMU sensors were attached to subject’s arm using velcro straps. One of them was placed on the posterior surface of wrist while the other one was located on the arm, approximately at 10 cm distance to the lateral epicondyle (Figure 3). Each IMU sensor was securely attached to the participant’s body with a self-adhering strap.

The procedure of application of the IMU-based system on the subject was performed by the same operator for all participants to the study. Then, subjects were prepared to execute the measurements of forward flexion, abduction, external and internal rotation at 90° of shoulder abduction. For each motion direction, participants were instructed to move the arm as far as they comfortably could, maintaining unchanged the initial arm position for the entire arc of movement. In addition, participants were required to perform shoulder movements at their self-selected speed [51,52]. Prior to measurements the correct execution of each maneuver was verbally explained and demonstrated by one of the investigators (I.N.). The subject was given some time to familiarize with the movement [53] until the researcher judged it to be correct.

Subsequently, the calibration procedure of the IMU-based system for a specific maneuver (e.g., forward flexion) was performed and data were gathered. Active shoulder movements were randomized across participants. Every participant was required to perform three repetitions of each maneuver. During each repetition two consecutive pairs of goniometer and IMU-based measurements were collected one after the other. For each pair of measurements, goniometer- and IMU-based assessments were performed simultaneously. In total, 6 goniometer and 6 IMU measurements were taken for each shoulder maneuver. Goniometer measurements were acquired by two raters (Medical Doctors). During each repetition one rater (E.J.C., RATER 1) made the first goniometer measurement and the second one (G.L., RATER 2), who was blind to the first measurement, repeated the same procedure.

IMU recordings were performed by two researchers (S.C. and L.B., IMU 1 and IMU 2, respectively) who were blind to goniometer measurements. Data were transmitted wirelessly to the gateway and subsequently processed by a specific software to calculate ROM. The RATER 1 and IMU 1 measurements, and similarly the RATER 2 and IMU 2 measurements, were acquired simultaneously. An operator (I.N.) assisted the subject to maintain the position of his/her arm to allow for consistency of measurements as well as effort relief for participant.

A rest interval was also given to participants between shoulder maneuvers to avoid fatigue effects [50,54,55]. The order of raters and researchers in collecting data was randomized among shoulder maneuvers and subjects.

#### 2.4.1. Participant Recruitment

Eight healthy control (mean age 44 ± 18 years, 1 female) and 8 CSCI patients (mean age 50 ± 12 years, 1 female) who had suffered traumatic spinal cord injuries were recruited in the study. Patients with CSCI were enrolled from the Spinal Unit of the Florence University Hospital between July and October 2020. The following inclusion criteria were adopted to select the sample:

- subjects over 18 years of age;
- C4–C7 cervical lesion level;
- at least one month post-injury;
- subjects with intact cognitive abilities;
- no joint contracture or severe spasticity in the affected upper limb (modified Ashworth scale greater than 3);
- sufficient Italian language skills.

The exclusion criteria were neuropsychiatric comorbidities and orthopedic impairments/or symptoms such as pain when moving their arm. The healthy controls were hospital staff or students from the University of Florence. Healthy individuals were all volunteers and they did not experience any type of shoulder disease. The demographics and clinical features of CSCI and healthy control groups are reported in Table 2.

#### 2.4.2. Ethical Consideration

All procedures were conducted according to the Declaration of Helsinki [56] and were approved by the Institutional Ethics Committee (Area Vasta Centro AOU Careggi, Florence, Italy—ref:17768_oss) following streamlined approval process for low-risk observational studies [57]. All Participants provided informed written consent (see the template in Supplementary Materials).

The wireless sensors (IMU) system does not need a direct interaction with the patient. The small size of the sensor and the stretch band where it is housed make it easily wearable and not bulky. These sensors are not equipped with additional actuators, such as buzzers, so they do not disturb the patient during the movement. Battery can last for more than two working days before a re-charge. The wireless connection assures that cables do not obstruct the patient and the doctor. Due to the restrictions imposed by Covid-19, the entire software development phase, including the first tests, was done entirely remotely. With regard to the experimental tests carried out at the Spinal Unit of Careggi Hospital in Florence, all protocols and provisions for the safety of patients and medical and university staff have been followed. It is important to stress that the experimental tests were performed with a smaller number of subjects than planned due to the anti-Covid protocol. Only 8 patients and 8 healthy individuals have participated to the experimental tests.

### 2.5. Metrics for Statistical Analysis

Concurrent validation of IMU-based sensor system for measuring the ROM active shoulder movements in CSCI patients and matched healthy control was tested by comparing the IMU system to the goniometer method. To test for concurrent validity, we evaluated the agreement between IMU-based ROM measurements and goniometer-based ROM measurements using Intraclass Correlation Coefficient (ICC) and Bland-Altman analysis, two methods that provide a measure of relative and absolute reliability of an instrument, respectively [58]. In addition, the degree of agreement between the measurements (Rater 1 versus Rater 2 and IMU 1 versus IMU 2 measurements, respectively) was assessed for every tested movement using ICC and Bland–Altman analysis.

A two-way random effect model, absolute agreement, multiple measurements ICCs (model 2, m) was used to test the inter-instrument and inter-tester reliability [59]. The guidelines for interpretation of ICC inter-rater agreement measurement proposed by Cicchetti [60] are exclusive for individual ICC, i.e., in this case ICC(2, 1). The Spearman-Brown formula [61,62] permits to evaluate the m-average ICC thresholds based on the individual ICC:

(1) $$\mathrm{ICC}\left(2,m\right)=\frac{m\cdot \mathrm{ICC}(2,1)}{1+(m-1)\cdot \mathrm{ICC}(2,1)}$$

In this work, ICC(2, 2) and ICC(2, 4) has been used. The thresholds calculated by Equation (1) are shown in Table 3 for equal to 1, 2 and 4.

Bland-Altman analysis quantifies the amount of agreement between two methods of measurement by constructing 95% limits of agreement (LOA), which provides an estimate of the interval where 95% of the differences between both methods fall [63,64] The 95% LOA is computed by using the mean difference ($\delta_{0}$) and the standard deviation (σ) of the differences between two measurements methods, and is defined by Carmona-Perez et al. in [41] as:

(2) $$\mathrm{LOA}=\delta_{0}\pm 1.96\cdot \sigma$$

The LOA in (2) defines two values: upper bound (UB) and low bound (LB). Criteria are required to assume acceptable agreement of two instruments [65]. As suggested by literature, acceptable agreement between measurements requires LOA to be within 10° of no difference between measurements [13]. However, as suggested in Mullaney’s study [66], when shoulder ROM measurements are taken by different raters using goniometer, LOA can be expected to be within 15° to consider acceptable agreement between raters. The sample size required to test the concurrent validity between IMU and goniometer methods was based on ICC.

Statistical analysis was performed in MATLAB${}^{©}$ and IBM SPSS Statistics software package (version 26).

## 3. Results

### 3.1. Accuracy of the IMU-Based System: Laboratory Tests

**Figure 4:**
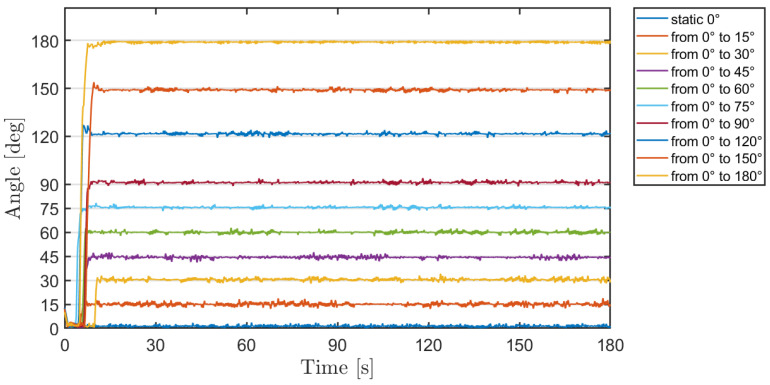
Results of experimental measurement campaign in laboratory.

The evaluation of the accuracy of the proposed IMU-based system has been carried out by testing it in our research laboratory. Static test was performed by mounting the IMU sensor on the arm of a goniometer. The accuracy test was executed by measuring the angle provided by the IMU sensor compared to the reference angle set with the goniometer. In particular, the goniometer moving arm was set and kept at a specific reference angle and data from IMU sensor was recorded for 3 min (see Figure 4). Ten different reference angles were tested, ranging from 0° to 180°. The reference angles and the IMU measurements are shown in Table 4, together with the average error. The mean difference of the IMU system and goniometer measurements was consistently below 3°, which indicated a quite good accuracy of the system in measuring the angle.

Both ICC and Bland-Altman analysis for the static test were performed and results are shown in Table 4. ICC mean value indicated an excellent agreement between IMU system and goniometer measurements. The electronic noise of IMU sensor can be evaluated in Table 4, where LOA between −3° and 5° indicates the noise influence in IMU measurements with the 95% of confidence interval.

### 3.2. Accuracy of IMU-Based and Goniometer Systems: Clinical Tests

Overall, ICCs for whole group of persons ranged from 0.94 to 0.97, indicating an excellent inter-rater reliability between the two raters and the two IMU measurements, as defined in Section 2.5. These four measurements were considered as four different proofs, for each of the arm movements (Table 5). When the groups were evaluated separately results showed different levels of reliability. In the CSCI group, an excellent agreement (ICCs ranging from 0.94 to 0.97) was confirmed for all tested movements. In healthy group, the agreement for flexion movement results fair (ICC of 0.84), whereas for the others movements the ICCs ranging from 0.94 to 0.97 was confirmed.

It is required to separate the measurements in different cluster to deeply investigate the reason of these assessment agreements identified by the analysis of the data in Table 5. The measurements have been divided in following clusters:

- Inter-instrument reliability and accuracy (See Section 3.2.1)
  - The two raters and the two IMUs measurements are considered as two different judges (Table 6),
  - RATER 1 and IMU 1 measurements are considered as two different judges (Table 7),
  - RATER 2 and IMU 2 measurements are considered as two different judges (Table 8).
- Inter-tester reliability and accuracy (See Section 3.2.2)
  - The two raters’ measurements are considered as two different judges (Table 9),
  - The two IMUs measurements are considered as two different judges (Table 10).

#### 3.2.1. IMU versus Goniometric Measurements

Table 6 displays the inter-instrument reliability through ICCs, mean differences and LOAs considering the two raters and the two IMUs measurements as two different judges. IMU-system and goniometer showed excellent agreement (ICCs ranging from 0.86 to 0.97, higher than threshold 0.85) for all tested movements in both groups with exception for flexion movements in healthy group, where the agreements between the two methods were fair (ICCs of 0.61).

In CSCI group mean differences between goniometer and IMU system were small for abduction and external rotation (bias of −1° and 1°, respectively), whereas larger values were observed for flexion and internal rotation (bias of 7° and −7°, respectively). In healthy group large mean differences were evidenced for flexion and abduction (bias of 4° and −6°, respectively). Lastly, LOAs were wide for both groups, consistently greater than 10° of the mean difference, indicating a non-homogeneus behaviour between goniometer and IMU method in measuring shoulder ROM for all tested movements, as explained in Section 2.5.

Additionally, in Table 7 and Table 8 is shown the inter-instrument reliability through ICCs, mean differences and LOAs when comparing each rater’s performance to the respective IMU measurement. Agreement between Rater 1 and IMU 1 measurements (Table 7), as Rater 2 and IMU 2 measurements (Table 8), were good to excellent for almost all movements in both groups (ICCs ranging from 0.77 to 0.98). Also, in healthy group a fair agreement was shown between Rater 1 and IMU 1 and Rater 2 and IMU 2 for flexion movement (ICC of 0.62), confirming the results shown in Table 6.

However, mean differences between Rater 2 and IMU 2 measurements were larger than those between Rater 1 and IMU 1 measurements in most of tested movements (Table 7 and Table 8). This trend was specifically evidenced for flexion and internal rotation in CSCI group and for flexion in healthy group. For flexion movement in CSCI group the mean difference between Rater 1 and IMU 1 measurements was found to be 1°, whereas the mean difference between the Rater 2 and IMU 2 measurements was 14°; this was also observed for internal rotation in which mean differences between rater 1 and IMU 1 measurements and rater 2 and IMU 2 measurements were −5° and −9°, respectively. Similarly, in healthy group for flexion movement rater 1 and IMU 1 measurements showed a mean difference of −2° and rater 2 and IMU 2 measurements showed a mean difference of 9°.

#### 3.2.2. Goniometer vs. Goniometer and IMU vs. IMU Measurements

Focusing on the raters measurements, ICCs for whole group ranged from 0.85 to 0.93, showing good to excellent inter-tester reliability between Rater 1 and Rater 2 measurements for all movements, as shown in Table 9. A differentiated analysis for each group indicated in CSCI group excellent agreement between raters for abduction and internal rotation (ICCs of 0.90 and 0.91, respectively) and good agreement for flexion and external rotation (ICCs of 0.85 and 0.83, respectively). In healthy group, excellent to good agreement was displayed for abduction, external and internal rotation (ICCs of 0.90, 0.84, and 0.84, respectively). A poor reliability (ICC of 0.47) was observed for the flexion movement, as depicted in Table 9.

However, mean differences between each raters’ measurements were quite large for almost all movements in both groups, with the highest value shown in CSCI group for the flexion movement (bias of −13°). In the healthy group the largest mean differences was shown for flexion and internal rotation (bias of −10° and 7°, respectively). Lastly, LOAs were wide for both groups, consistently greater than 15° of the mean difference, indicating a non-homogeneus behaviour between Rater 1 and Rater 2 in measuring shoulder ROM for all tested movements.

Focusing on the IMUs measurements, ICCs for whole group ranged from 0.988 to 0.999, showing very excellent inter-tester reliability between IMU 1 and IMU 2 measurements for all movements, as depicted in Table 10. Very excellent inter-tester reliability were confirmed also when CSCI and healthy groups were separately analyzed (ICCs ranging from 0.979 to 0.998).

In addition, mean differences were found very small for all tested movements in CSCI group, ranging from −1° to 1°, as well as in healthy group, ranging from −1° to 2°. Lastly, LOAs were narrow, very similar to those found in the preliminary laboratory tests, and consistently showed that the difference in IMU 1 and IMU 2 measurements was within 10° of the mean difference for approximately 95% of participants in both group.

## 4. Discussion

This is the first study to evaluate the validity of wireless wearable IMU-based sensors system for the assessment of shoulder ROM in patients with CSCI in clinical environment. A custom IMU-based system was developed and compared to the method used currently in clinic, i.e., a sanitary operator measuring the ROM with a goniometer. The good accuracy of the IMU-based system was proven in laboratory tests with absolute error consistently below 3° and 95% LOA within 10°. Together with the accuracy of the proposed system, we aimed to provide a measure of the accordance between the two measuring methods: the IMU-based and the rater-based method. According to the results, the concurrent validity of IMU system was partially confirmed. ICCs values were found to be very high for all tested shoulder maneuvers except for flexion in healthy group, showing an excellent relative validity of the IMU system. However, given that the mean differences between IMU and goniometer measures were shown to be relatively high for the majority of movements alongside a high distance between LOAs, the absolute validity of the IMU system is not strongly supported. The two methods might not seem interchangeable. In addition, while two raters’ measurements showed a considerably low inter-rater reliability, IMU system was proven to have excellent agreement when compared one measurement to the other.

Within limited literature regarding the concurrent validation of IMU sensors against goniometer in assessing shoulder ROM, we found little similarities with other studies though the differences in research methodology may interfere with comparisons. Yoon et al. [37] assessed the validity of IMU sensors in evaluating upper extremity movements while passively positioning subjects’ shoulder at specific angles using a goniometer (shoulder flexion 0°–170°, abduction 0°–170°, external rotation 0°–90°, and internal rotation 0°–60° angles). The authors showed large mean bias and a high distance between LOA for most of shoulder position except for flexion (0–135°), abduction (0°), external (0°) and internal rotation (0°–45°), though the relative reliability between goniometer and IMU by means of ICC analysis was not evaluated. In a more recent study, Rigoni and coworkers [13] validated the IMU system by testing active shoulder ROM on healthy subjects and, similar to our study, showed high ICC values for all tested movements (flexion, abduction, external and internal rotation at 90 shoulder abduction). However, they showed absolutely reliability of IMU system through very small mean bias (approximately 0°) and narrow LOA (−4.5° to 3.2°).

Our study showed excellent relative reliability of IMU system in comparison with goniometer method in most movements performed by CSCI and healthy groups. However, some high ICC values in our results should be considered with caution as it is known that the ICC values are influenced by the range of measured values (sample heterogeneity) [66]. Higher ranges (greater heterogeneity) are associated with higher ICCs, independent of actual measurement error [57,58]. In fact, in some movements where we obtained very high ICC values, there was also found a high variability of the data. For instance, in CSCI group for internal rotation the standard deviation was about 30% of the mean value of ROM. This can mask poor trial-to-trial consistency and not accurately represent the high agreement of measurements between the two methods. These interpretations of ICCs are in conformity with previous studies using IMU sensors to evaluate motion in disease of motor control [41,67].

On the contrary, our study revealed the existence of measurement differences between IMU system and goniometer in CSCI patients with 95% LOA for the two instruments ranged from −22° to 32°, showing a certain change in measuring between goniometer and IMU for all tested movements. These wider LOA were confirmed also for control group of healthy subjects (LOA ranged from −20° to 28°).

Several factors regarding the setup in our study might have influenced the results by IMU system measurements. The exclusion of magnetometer usage in our IMU sensors could be the first possible reason. This choice allowed to avoid local magnetic disturbances which could affect IMUs accuracy due to magnetometers sensitivity to the magnetic fields [68,69,70,71]. In our clinical tests the hospital environment and electronic controls of motorized wheelchair could influence the efficiency of magnetometer. The downside of this decision, on the other hand, is that an IMU system without a magnetometer could have provided lower accuracy in orientation estimation, specifically while testing a mobile joint like shoulder with several degrees of freedom due to bony constraint scarcity and soft tissue function [72]. Another possible source of measurement error for our IMU system could be the presence of wheelchair in the setup used to test CSCI patients, which seems to interfere in performing motion in one single plane specifically for flexion and abduction movements. Indeed, a previous study by Lee and colleagues [39], in which the validity of Kinetec against goniometer was tested in measuring active and passive shoulder ROM movements, showed that the deviation from planes of unrestricted motion was an important source in increasing 95% limit of agreement between two measurement methods. Moreover, for maneuvers like external and internal rotation with forearm abduction at 90° an additional potential cause for discrepancies of measurements between goniometer and IMU system includes differences in starting positions of the forearm among trials. In fact, even with the assistance of an operator, holding a perfect position of forearm parallel to the floor by the subject was not completely guaranteed and it could provoke differences in measurement starting point between IMU system and raters who were instructed to maintain the stationary arm of goniometer fixed consistently parallel to the floor (see Table 1 for goniometer landmarks).

Additionally, when a deeper analysis comparing each rater’s performance to the respective IMU measurement was executed, larger differences of measurements (mean bias) were found between Rater 2 and IMU 2 than between Rater 1 and IMU 1, especially for flexion and internal rotation movements (see Table 7 and Table 8). Moreover, inter-tester reliability for IMU measurements showed perfect accordance between IMU 1 and IMU 2 (ICCs from 0.979 to 0.998; mean bias from −1° to 2°; and LOA within 10°) for both groups (see Table 10), whereas inter-tester reliability for goniometer measurements evidenced larger differences (mean bias from −13° to 7°; LOA wider than 15°) between Rater 1 and Rater 2, even though ICC values appeared to be good to excellent except for flexion in healthy group (see Table 9). Thus, it is possible to assume that inconsistency between raters partially contributes to the differences observed in measuring shoulder ROM between IMU and goniometer. This also confirmed similar findings from previous literature which evaluated inter-rater reliability of goniometer measurements for shoulder ROM and showed that reliability of goniometer is lower when measurements are taken by different raters [43].

Furthermore, concerning the inter-tester reliability between two IMU measurements, Rigoni and coworkers [13] found low agreement between measurements when one goniometer and one IMU measurement were taken by each assessor for each movement. They suggested that IMU measurements recorded by one assessor could not be exchanged for another one’s measurements. Differently, as we conducted three repetition for each tested movement and recorded pairs of two goniometer- and two IMU-based measurements simultaneously during every repetition, a very high inter-tester reliability between two IMU measurements was found. This implicates that differences of IMU measurements between assessors found in Rigoni’s study might be ascribed to the inconsistency of movements performed over time rather than the assessor’s performance. This could suggest that, despite its level of accuracy, our IMU system is a stable and operator-independent method to measure shoulder ROM in patients with CSCI.

Altogether, our custom IMU-based system was shown to be a promising tool to assess shoulder ROM in CSCI patients according to several benefits. First, IMU could be introduced in clinical setting as a stable tool which is independent from assessor’s availability and manual skills. Second, the simplicity of setup could allow the easy self-applying procedure of IMU system and increase the variety of body contexts in which it is possible to collect measurements. Third, IMU-based system could allow clinicians to remotely monitor patients’ movements while these latter staying out of routine clinical system (e.g., house), and thus make the caring more convenient and economical in such patients with motor difficulties by easy accessibility [73,74,75]. Finally, IMU system could also provide patients with feedback on their performance and progress during rehabilitation [13].

Despite the promising findings, the current study showed some limitations, which could suggest future research. The setup of IMU system without magnetometer might have affected the accuracy in measuring active unrestricted movements of shoulder. The magnetometer integration on IMU system could be introduced in future studies due to the fact that it is essential to evaluate the possible implementation of IMU system for distant monitoring of patients. In addition, the number of participants was relatively small which did not allow us to detect statistically significant differences between CSCI and healthy groups in each shoulder ROM via IMU system. Furthermore, the current study focused only on the evaluation of ROM of simple movements, which limits the applicability of the results to more complex and functional tasks which are more identical to daily-life activities (e.g., reach-to-grasp upper limb movements) [40]. Finally, our study did not analyze dynamic movement characteristics of upper limbs, including angular velocity and acceleration, that could provide a more comprehensive clinical assessment of patients [40]. All these factors should be considered in future studies on assessing the validity of IMU system [64].

## 5. Conclusions

This work aimed to provide a methodological study on the validity of a customized wireless wearable IMU-based sensors system to measure the shoulder ROM in patients with cervical spinal cord injury. Laboratory and clinical trials have been performed to evaluate the accuracy of the IMU-based system, and to compare it to the goniometer-based measurements taken by sanitary operators. The results showed an excellent relative reliability between the IMU-based system and goniometer-operator method, even though the two methods might not seem interchangeable. Nonetheless, unlike the goniometer method, the accuracy of IMU system is not influenced by the operator who carries on the measurement. Therefore, the proposed system can be a potential tool to be integrated in clinical settings for monitoring shoulder ROM in patients with cervical spinal cord injury.

It is worth mentioning that, in addition to static angles, the IMU sensors also capture dynamic kinematic parameters (e.g., angular velocity and acceleration) that could be used with these patients to quantify muscle stress and effort during movements by estimating fatigue-related tremor. Real-time measurements from the sensors could be further used to provide feedback to the patient during the execution of motions in physical therapy.

Furthermore, our validation work poses the base for a possible use of the IMU system for a remote monitoring of patients at home, and this feature could be crucial, in particular during emergency situations such as COVID-19 pandemic. In fact, a wireless wearable IMU system automatically could collect data on the movements of patient’s arms and allow us to create a database with all the performance.

Possible future developments of the proposed system span from the inclusion of the magnetometer measure to improve the accuracy, to the fusion of the kinematics data to provide a deeper interpretation in quantifying motion recovery status of patient during the rehabilitation process, and, finally, to the use of artificial intelligence (AI) to automatically manage the everyday activities of the patient.

## Figures and Tables

**Table 1:** Movement and Goniometer Landmarks.

Shoulder Movement	Goniometer Landmarks		
	Center Fulcrum	Stationary Arm	Moving Arm
**Flexion**	Lateral aspect of the glenohumeral joint	Parallel to the midline of the trunk	Lateral epicondyle of the humerus
**Abduction**	Posterior aspect of the glenohumeral joint	Laterally along the trunk, parallel to the spine.	Lateral epicondyle of the humerus
**External Rotation at 90° abduction**	Olecranon process ulna	Parallel to the floor	Ulna styloid process
**Internal Rotation at 90° abduction**	Olecranon process ulna	Parallel to the floor	Ulna styloid process

**Table 2:** Characteristics of the participants to the experimental tests. Abbreviations used: P stands for patient; HC stands for healthy control; R stands for Right; L stands for Left; AIS stands for American Spinal Injury Association Impairment Scale; T stands for traumatic; SURG stands for Surgery Intervention; BTOX stands for Botulin Toxin Intervention.

Patients	Sex	Age (Years)	Dominant Arm	Lesion Level	AIS Grade	Etiology	Severity of Lesion	Time since Injury (Years)	Shoulder Intervention
P1	Male	56	R	C4	D	T	Incomplete	1	/
P2	Male	63	R	C6–C7	C	T	Incomplete	2	/
P3	Male	63	R	C5–C6	B	T	Incomplete	5	SURG
P4	Male	32	R	C4	D	T	Incomplete	1	BTOX
P5	Male	52	L	C6	D	T	Incomplete	2	/
P6	Male	43	R	C6–C7	A	T	Complete	21	BTOX
P7	Male	59	R	C4	D	T	Incomplete	5	SURG
P8	Female	34	R	C4–C5	D	T	Incomplete	3	/
HC1	Male	65	R						
HC2	Female	27	R						
HC3	Male	27	R						
HC4	Male	34	L						
HC5	Male	49	R						
HC6	Male	36	R						
HC7	Male	37	R						
HC8	Male	76	R						

**Table 3:** Thresholds for interpretation of Intraclass Correlation Coefficient (ICC) between measurements.

Reliability	ICC(2, 1)	ICC(2, m)	
		m = 2	m = 4
Poor	<0.4	<0.57	<0.73
Fair	0.4–0.6	0.57–0.75	0.73–0.86
Good	0.6–0.75	0.75–0.85	0.86–0.92
Excellent	>0.75	>0.85	>0.92

**Table 4:** Main statistical results of the laboratory measurement campaign.

Goniometer	IMUs Average (σ)	Difference	ICC (95% CI)	LOA
0°	1.45° (0.77°)	1.45°	0.9996 (0.9994; 0.9997)	−3.19°; 4.92°
15°	15.19° (0.96°)	0.19°		
30°	30.90° (0.94°)	0.90°		
45°	45.23° (1.19°)	0.23°		
60°	61.51° (1.64°)	1.51°		
75°	76.11° (0.82°)	1.11°		
90°	92.06° (1.16°)	2.06°		
120°	122.74° (1.42°)	2.74°		
150°	151.40° (2.56°)	1.40°		
180°	177.02° (2.00°)	2.98°		

Abbreviations: *σ* stands for standard deviation; ICC stands for Intraclass Correlation Coefficient; CI stands for Confidence Interval (95% for this work); LOA stands for Limits of Agreement.

**Table 5:** Inter-rater reliability indicators for two raters and two inertial measurement units (IMUs) measurements as four different judges, it calculated as ICC(2, m) with m = 4 and the confidence interval is 95%.

	Whole Group n = 48			CSCI Group n = 24			Healthy Group n = 24		
	**ICC(2, m)**	**LB**	**UB**	**ICC(2, m)**	**LB**	**UB**	**ICC(2, m)**	**LB**	**UB**
Flexion	0.94	0.86	0.97	0.94	0.82	0.98	0.84	0.65	0.93
Abduction	0.97	0.96	0.98	0.97	0.95	0.99	0.94	0.88	0.97
External Rotation	0.97	0.95	0.98	0.95	0.91	0.98	0.97	0.94	0.98
Internal Rotation	0.95	0.91	0.97	0.96	0.90	0.98	0.94	0.86	0.97

Abbreviations: LB: Lower Bound, UB: Upper Bound; n, measurements per method.

**Table 6:** Inter-instrument reliability indicators for goniometer and IMU measurements in the whole group, cervical spinal cord injury (CSCI) patients and healthy group.

	Goniometer Average (σ)	IMUs Average (σ)	Difference (σ)	ICC (95% CI)	LOA
Whole group (n = 96)					
Flexion	140° (18°)	134° (20°)	6° (12°)	0.86 (0.75; 0.92)	−19°; 30°
Abduction	146° (20°)	149° (21°)	−3° (8°)	0.95 (0.91; 0.97)	−19°; 13°
External Rotation	79° (16°)	78° (18°)	1° (8°)	0.94 (0.91; 0.96)	−15°; 16°
Internal Rotation	53° (14°)	56° (13°)	−3° (8°)	0.90 (0.81; 0.94)	−19°; 12°
CSCI group (n = 48)					
Flexion	131° (20°)	124° (20°)	7° (13°)	0.86 (0.65; 0.93)	−17°; 32°
Abduction	135° (20°)	136° (19°)	−1° (9°)	0.95 (0.91; 0.97)	−18°; 15°
External Rotation	71° (13°)	70° (18°)	1° (10°)	0.88 (0.78; 0.93)	−19°; 20°
Internal Rotation	51° (16°)	58° (16°)	−7° (8°)	0.90 (0.55; 0.96)	−22°; 8°
Healthy group (n = 48)					
Flexion	149° (9°)	145° (14°)	4° (12°)	0.61 (0.31; 0.78)	−20°; 28°
Abduction	156° (13°)	162° (13°)	−6° (7°)	0.87 (0.53; 0.95)	−20°; 9°
External Rotation	87° (14°)	86° (14°)	1° (5°)	0.97 (0.94; 0.98)	−9°; 11°
Internal Rotation	55° (12°)	55° (10°)	0° (7°)	0.89 (0.81; 0.94)	−14°; 13°

Abbreviations: IMU, Inertial Measurement Unit; ICC, Intraclass Correlation Coefficient; CI, confidence interval; LOA, Limit of Agreement; n, measurements per method; *σ*, Standard deviation.

**Table 7:** Inter-instrument reliability indicators for goniometer and IMU measurements in CSCI patients and healthy group for RATER 1 and IMU 1.

	Goniometer Average (σ) RATER 1	IMUs Average (σ) IMU 1	Difference (σ)	ICC (95% CI)	LOA
Whole group (n = 48)					
Flexion	134° (16°)	135° (21°)	0° (11°)	0.90 (0.83; 0.95)	−22°; 21°
Abduction	144° (18°)	148° (21°)	−5° (8°)	0.94 (0.86; 0.97)	−20°; 12°
External Rotation	77° (13°)	78° (17°)	−2° (10°)	0.89 (0.80; 0.94)	−21°; 17°
Internal Rotation	56° (13°)	57° (13°)	−1° (9°)	0.89 (0.80; 0.94)	−18°; 15°
CSCI group (n = 24)					
Flexion	124° (18°)	124° (20°)	1° (11°)	0.92 (0.81; 0.96)	−20°; 22°
Abduction	133° (17°)	136° (19°)	−3° (8°)	0.94 (0.85; 0.97)	−20°; 14°
External Rotation	68° (11°)	71° (18°)	−2° (13°)	0.77 (0.49; 0.90)	−27°; 22°
Internal Rotation	53° (15°)	58° (16°)	−5° (8°)	0.91 (0.72; 0.96)	−21°; 11°
Healthy group (n = 24)					
Flexion	144° (7°)	146° (14°)	−2° (12°)	0.62 (0.10; 0.83)	−24°; 21°
Abduction	156° (11°)	161° (13°)	−6° (8°)	0.81 (0.33; 0.93)	3°; 22°
External Rotation	85° (9°)	86° (13°)	−1° (5°)	0.94 (0.86; 0.97)	−12°; 9°
Internal Rotation	58° (11°)	56° (10°)	2° (7°)	0.85 (0.66; 0.94)	−12°; 17°

Abbreviations: IMU, Inertial Measurement Unit; ICC, Intraclass Correlation Coefficient; CI, confidence interval; LOA, Limit of Agreement; n, measurements per method; *σ*, Standard deviation.

**Table 8:** Inter-instrument reliability indicators for goniometer and IMU measurements in CSCI patients and healthy group for RATER 2 and IMU 2.

	Goniometer Average (σ) RATER 2	IMUs Average (σ) IMU 2	Difference (σ)	ICC (95% CI)	LOA
Whole group (n = 48)					
Flexion	146° (18°)	134° (20°)	12° (11°)	0.83 (0.09; 0.94)	−10°; 33°
Abduction	147° (21°)	149° (21°)	−2° (8°)	0.96 (0.93; 0.98)	−17°; 14°
External Rotation	81° (18°)	78° (19°)	3° (5°)	0.97 (0.92; 0.99)	−6°; 13°
Internal Rotation	50° (15°)	56° (14°)	−6° (6°)	0.91 (0.56; 0.97)	−18°; 7°
CSCI group (n = 24)					
Flexion	137° (21°)	123° (20°)	14° (11°)	0.82 (−0.15; 0.95)	−7°; 35°
Abduction	137° (22°)	136° (20°)	1° (8°)	0.96 (0.91; 0.98)	−15°; 17°
External Rotation	73° (14°)	69° (18°)	4° (6°)	0.96 (0.85; 0.98)	−8°; 15°
Internal Rotation	49° (18°)	58° (16°)	−9° (6°)	0.91 (0.08; 0.98)	−21°; 4°
Healthy group (n = 24)					
Flexion	154° (8°)	145° (14°)	9° (11°)	0.62 (−0.12; 0.85)	−12°; 30°
Abduction	157° (15°)	162° (13°)	−5° (7°)	0.91 (0.64; 0.97)	−18°; 8°
External Rotation	89° (17°)	87° (15°)	3° (4°)	0.98 (0.92; 0.99)	−5°; 10°
Internal Rotation	51° (11°)	54° (10°)	−3° (5°)	0.93 (0.79; 0.97)	−13°; 7°

Abbreviations: IMU, Inertial Measurement Unit; ICC, Intraclass Correlation Coefficient; CI, confidence interval; LOA, Limit of Agreement; n, measurements per method; *σ*, Standard deviation.

**Table 9:** Inter-tester/rater reliability indicators for goniometer measurements in CSCI patients and healthy group for RATER 1 and RATER 2.

	Goniometer Average (σ) RATER 1	Goniometer Average (σ) RATER 2	Difference (σ)	ICC (95% CI)	LOA
Whole group (n = 48)					
Flexion	134° (16°)	146° (18°)	−12° (8°)	0.85 (−0.15; 0.96)	−26°; 3°
Abduction	144° (18°)	147° (21°)	−3° (10°)	0.93 (0.88; 0.96)	−22°; 16°
External Rotation	77° (13°)	81° (18°)	−5° (9°)	0.89 (0.75; 0.94)	−23°; 13°
Internal Rotation	56° (13°)	50° (15°)	5° (8°)	0.89 (0.62; 0.95)	−9°; 20°
CSCI group (n = 24)					
Flexion	124° (18°)	137° (21°)	−13° (8°)	0.85 (−0.16; 0.96)	−29°; 3°
Abduction	133° (17°)	137° (22°)	−4° (11°)	0.90 (0.77; 0.96)	−27°; 18°
External Rotation	68° (11°)	73° (14°)	−5° (9°)	0.83 (0.55; 0.93)	−22°; 12°
Internal Rotation	53° (15°)	49° (18°)	4° (9°)	0.91 (0.78; 0.97)	−13°; 21°
Healthy group (n = 24)					
Flexion	144° (7°)	154° (8°)	−10° (6°)	0.47 (−0.30; 0.81)	−23°; 2°
Abduction	156° (11°)	157° (15°)	−1° (8°)	0.90 (0.77; 0.96)	−16°; 14°
External Rotation	85° (9°)	89° (17°)	−4° (10°)	0.84 (0.61; 0.93)	−24°; 15°
Internal Rotation	58° (11°)	51° (11°)	7° (6°)	0.84 (0.04; 0.95)	−5°; 19°

Abbreviations: ICC, Intraclass Correlation Coefficient; CI, confidence interval; LOA, Limit of Agreement; n, measurements per method; *σ*, Standard deviation.

**Table 10:** Inter-tester/rater reliability indicators for IMU measurements in CSCI patients and healthy group for IMU 1 and IMU 2.

	IMUs Average (σ) IMU 1	IMUs Average (σ) IMU 2	Difference (σ)	ICC (95% CI)	LOA
Whole group (n = 48)					
Flexion	135° (21°)	134° (20°)	0° (2°)	0.997 (0.995; 0.998)	−4°; 5°
Abduction	149° (21°)	149° (21°)	0° (2°)	0.999 (0.998; 0.999)	−3°; 3°
External Rotation	78° (17°)	78° (19°)	0° (4°)	0.988 (0.978; 0.993)	−7°; 8°
Internal Rotation	57° (13°)	56° (14°)	1° (2°)	0.993 (0.983; 0.996)	−3°; 5°
CSCI group (n = 24)					
Flexion	124° (20°)	123° (20°)	1° (2°)	0.997 (0.993; 0.999)	−4°; 5°
Abduction	136° (19°)	136° (19°)	−1° (2°)	0.998 (0.994; 0.999)	−4°; 3°
External Rotation	71° (18°)	69° (18°)	1° (4°)	0.988 (0.972; 0.995)	−6°; 9°
Internal Rotation	58° (16°)	58° (16°)	0° (2°)	0.996 (0.990; 0.998)	−4°; 5°
Healthy group (n = 24)					
Flexion	146° (14°)	145° (14°)	0° (2°)	0.995 (0.988; 0.998)	−4°; 5°
Abduction	162° (13°)	162° (13°)	0° (1°)	0.998 (0.995; 0.999)	−2°; 2°
External Rotation	86° (13°)	87° (15°)	−1° (4°)	0.979 (0.952; 0.991)	−8°; 7°
Internal Rotation	56° (10°)	54° (10°)	2° (2°)	0.985 (0.852; 0.996)	−2°; 5°

Abbreviations: IMU, Inertial Measurement Unit; ICC, Intraclass Correlation Coefficient; CI, confidence interval; LOA, Limit of Agreement; n, measurements per method; *σ*, Standard deviation.

**Figure A1:**
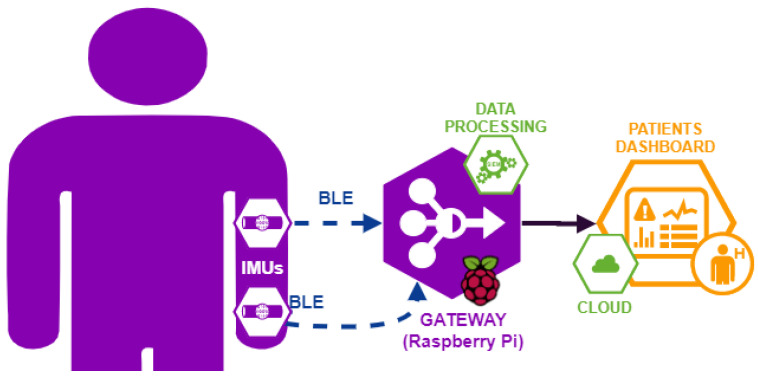
Scheme of the Hardware Module of the system. In purple, the components of system hardware module; in green, the components of system software module and in orange, the components of data aggregation/visualization module.

**Figure A2:**
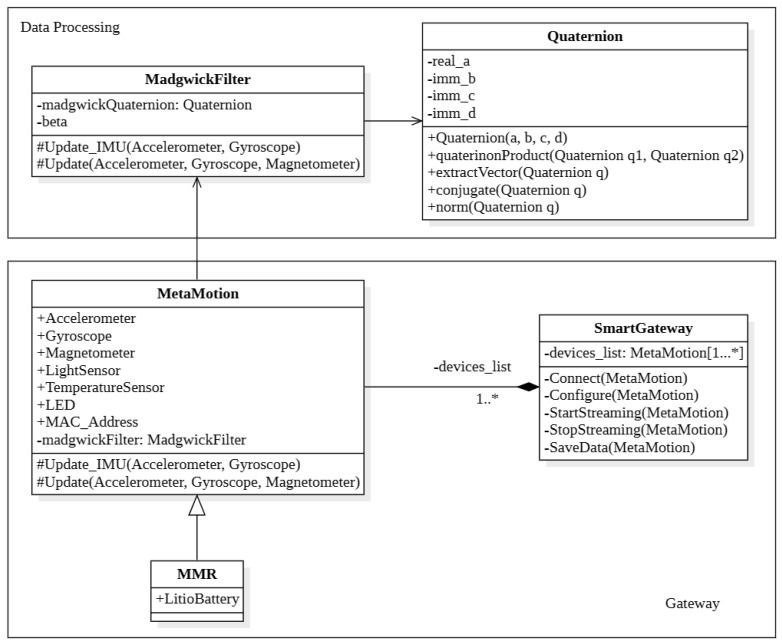
UML Class Diagram of the back-end *software module* of IMU sensors network system used in this work. The writing (1...*) stands for “from 1 to infinity”.

## Data Availability

The data presented in this study are available on request from the corresponding author. The data are not publicly available due to privacy issue.

## Appendix A. Technical Information about Wearable Sensors System for Motion Assessment

### Appendix A.1. System Hardware Architecture

The Hardware system is composed of commercial IMU sensors, which are connected to a gateway (Raspberry Pi). The two sensors are positioned one on the arm and one on the wrist of the individual (Figure A1). The Bluetooth Low Energy (BLE) is the communication protocol used to transmit data from sensor to the gateway, in compliance with standard IEEE 802.15.6, typically used in Wireless Body Area Networks (WBANs). Once data are received by the gateway, they are processed by a custom application and stored on a cloud database.

Among the many IMU sensors on the market, the MbientLab MetaMotion R [76] was selected, since it shows sufficient accuracy for this application (from datasheet [76], the accuracy is less than 1 degree RMS), and it is characterized by small dimensions and low cost, compared to other similar sensors. The accuracy of IMU-based system has been verified in Section 3.1.

The manufacturer provides a mobile application, which does not include synchronization functionality between multiple sensors, thus we had to implement a customized gateway for the synchronization of sensors, the collection and the analysis of the data.

### Appendix A.2. Data Processing and ROM Calculation with IMU-Based System

*Notation:* Scalar variables are displayed in sans serif, lowercase letters; their realizations in serif, italic fonts, *x*. Vectors and matrices are denoted by bold lowercase and uppercase letters, respectively, e.g., x indicates a vector and X indicates a matrix. Quaternions are denoted by bold lowercase letters with tilde sign as a hat $\tilde{x}$. Coordinate frame are displayed in sans serif, italic fonts, uppercase letters *X*. A pre-subscript denotes the source coordinate frame and a pre-superscript denotes the destination coordinate frame, ${}_{A}^{B}\tilde{x}$ denoted a quaternion from coordinate frame *A* to *B*. In the case that only a pre-superscript is present in the quaternion, it means that the quaternion is measured and represented in the same coordinate frame ${}^{B}\tilde{x}$.

The range of motion (ROM) can be calculated by using accelerometer or gyroscope measurements, both present in the IMU sensors. A fusion of those data can enhance the accuracy of the ROM calculation. This fusion is provided by the Madgwick filtering [77]. In this study, the monitoring of the arm movement has been done in the vertical plane. For this reason, the fusion of the data coming from the accelerometer and the gyroscope by using the Madgwick filter algorithm [77] is assumed to be enough for ROM assessment.

Typically, to represent orientations and rotations of generic object in three dimensions, two different mathematical notations are used: Euler angles or quaternions. Usually, data fusion filter algorithms use quaternions due to lower computational cost than Euler angles. A quaternion is a mathematical iper-complex entity ($\tilde{q}\in C^{4}$), composed of a scalar part and a vector part. A quaternion is generally represented as

(A1) $$\tilde{q}=a+bi+cj+dk$$

where *a*, *b*, *c* and *d* are real numbers and i, j, k are orthogonal versors.

Denoting with u and α the rotation axis and angle, respectively, Equation (A1) can be rewritten as

(A2) $$\tilde{q}=\cos\frac{\alpha }{2}+u\sin\frac{\alpha }{2}$$

where u is described by the versors i, j, k.

The Madgwick filter algorithm is deployed in the Raspberry Pi module to reduce the centrifugal acceleration component and to reduce the drift error due to the angular velocity (gyroscope) integration. The Madgwick filtering is based on the estimated quaternion ${}_{W}^{I}{\tilde{q}}_{est,t+1}$ from fixed global coordinate (*W*) to inertial coordinate (*I*), given the accelerometer measure, the gyroscope measure and the estimated quaternion ${}_{W}^{I}{\tilde{q}}_{est,t}$ at previous step time *t*

(A3) $${}_{W}^{I}{\tilde{q}}_{est,t+1}=\int_{t}^{t+1}\left(\underset{\mathrm{Gyroscope}\;\mathrm{component}}{\underbrace{\frac{1}{2}{}_{W}^{I}{\tilde{q}}_{est,t}⊗\left[0,{}^{I}\omega_{t+1}\right]}}-\beta \underset{\mathrm{Accelerometer}\;\mathrm{component}}{\underbrace{\frac{\nabla f({}_{W}^{I}{\tilde{q}}_{est,t},{}^{W}\tilde{g},{}^{I}{\tilde{a}}_{t+1})}{\left\|\nabla f({}_{W}^{I}{\tilde{q}}_{est,t},{}^{W}\tilde{g},{}^{I}{\tilde{a}}_{t+1})\right\|}}}\right)\cdot dt$$

where

- β is a parameter which weights the accelerometer contribution on the quaternion estimate;
- ⊗ indicates quaternions product;
- ∇f(·) indicates an error direction on the solution surface defined by the objective function, $f({}_{W}^{I}{\tilde{q}}_{est,t},{}^{W}\tilde{g},{}^{I}{\tilde{a}}_{t+1})$ (function defined as in (A4)) and its Jacobian;
- ‖∇f(·)‖ indicates a norm of function $\nabla f({}_{W}^{I}{\tilde{q}}_{est,t},{}^{W}\tilde{g},{}^{I}{\tilde{a}}_{t+1})$;
- ω is conversion from gyroscope 3D measurements into a quaternion;
- $\tilde{g}$ is conversion from gravity 3D vector into a quaternion;
- $\tilde{a}$ is conversion from accelerometer 3D measurements into a quaternion;

The gyroscope component in Equation (A3) represents the quaternion that describes the rate of change of the earth frame (inertial frame of reference with the gravity vector as the vertical) relative to sensor frame based on gyroscope measurements, ${}^{I}\omega_{t+1}$. The acceleration component in Equation (A3) represents the error correction function based on orientation increment given by the accelerometer measurements, ${}^{I}{\tilde{a}}_{t+1}=\left[0,a_{t+1}\right]$, compared to gravity component, ${}^{W}\tilde{g}=\left[0,0,0,1\right]$. In fact, the term $f({}_{W}^{I}{\tilde{q}}_{est,t},{}^{W}\tilde{g},{}^{I}{\tilde{a}}_{t+1})$ is defined as

(A4) $$f\left({}_{W}^{I}{\tilde{q}}_{est,t},{}^{W}\tilde{g},{}^{I}{\tilde{a}}_{t+1}\right)={}_{W}^{I}{\tilde{q}}_{est,t}^{*}⊗{}^{W}\tilde{g}⊗{}_{W}^{I}{\tilde{q}}_{est,t}-{}^{I}{\tilde{a}}_{t+1}$$

where ${}_{W}^{I}{\tilde{q}}_{est,t}^{*}$ indicates a conjugate quaternion of ${}_{W}^{I}{\tilde{q}}_{est,t}$

For further details about these formulas, their full proofs and explanations, see [77].

The procedure to evaluate the angle of motion with the IMU-based system begins with a calibration procedure, in which the sensor has been positioned with its y-axis along the arm, i.e., the y-axis in sensor coordinate frame corresponds to one principal axis of inertia in arm coordinate frame. The initial position of the arm defines a versor ($v_{0}$), which has been saved before the begin of every movements.

In the initial position, the arm has been positioning in vertical position to reduce the influence of gyroscope drift. During the test at every display refresh time (50 frames per seconds, so 0.02 s), the arm position defines a versor (v) and the developed gateway shows in a monitor the angle (θ) between $v_{0}$ and v, calculated by:

(A5) $$\theta =\arccos\left(\langle v,v_{0}\rangle \right)$$

where $\langle v,v_{0}\rangle$ indicates scalar product between versors v and $v_{0}$.

### Appendix A.3. System Software Architecture

A custom Python software was deployed to connect multiple sensors simultaneously, to synchronize the sensor readings, and to send data to the gateway for storing and processing. The general scheme of the software is represented by using the Unified Modeling Language (UML) in Figure A2 and it is composed of:

- **MadgwickFilter** represents the mathematical tool to update quaternion value from accelerometer and gyroscope measurements. It has two main attributes:
  - *madgwickQuaternion* is an instance of the Quaternion class. It is modified every times is called the function *update* using Equation (A4) to evaluate the angle measured by each sensor.
  - *beta* is the editable parameter to change the accelerometer contribution weight, as shown in Equation (A3).
- **Quaternion** is the implemented mathematical library to help with operations with quaternions, e.g., product between quaternions, conjugate, apply rotation to vector, etc. This mathematical library is been used by MadgwickFilter class to update its quaternion.
- **MetaMotion** is the main class of the schema in Figure A2, it represents an abstraction of the sensors used for this work. As these devices use the Bluetooth protocol to communicate, they are uniquely identified by their MAC address. The sensor unit has different sensors, in particular accelerometer and gyroscope, used for this work [76].
- **SmartGateway (*****Raspberry*****)** is the back end unit of data (post) processing. This component is in charge of synchronizing the connection with the various devices, which may be of different types. It has to connect with these devices, configure them by setting the date rate parameters, and allow us to broadcast the data stream.
  The angle of the arm movement has been calculated every display refresh, because the data acquisition rate of these devices is not time-constant. When the movement is finished, this unit has to collect and store all data into his internal storage: this operation has been implemented to analyze and compare data of past movements with the actual.

In Figure A2 both the data visualization part (the orange module of Figure 2) and the cloud database part (the green hexagonal icon with the cloud symbol inside, both in Figure 2 and Figure A1) are missing: these two parts are currently under development. The priority has been given to the implementation of the sensor synchronization and data processing part of the system, due to only these parts are necessary for this work.
